# Cord Blood Platelet-Rich Plasma in Cesarean Section Wound Management

**DOI:** 10.1155/ogi/4155779

**Published:** 2024-12-12

**Authors:** Amornrat Thanachaiviwat, Sutham Suthaporn, Patana Teng-umnuay

**Affiliations:** ^1^Department of Obstetrics and Gynaecology, Division of Obstetrics and Gynaecology, Police General Hospital, Bangkok, Thailand; ^2^Department of Integrative Medicine, College of Integrative Medicine, Dhurakij Pundit University, Bangkok, Thailand

**Keywords:** cord blood platelet-rich plasma, platelet-rich plasma, wound healing

## Abstract

Platelet-rich plasma (PRP) promotes the wound-healing process and reduces pain. Cord blood platelet-rich plasma (CB-PRP), which can be easily collected from the umbilical cord and reapplied to a cesarean section wound, has been proposed to have similar effects as PRP. This paper hypothesizes that CB-PRB would provide beneficial effects in terms of wound healing and pain reduction in women undergoing cesarean section. This study is a randomized controlled trial involving 52 pregnant women who underwent cesarean sections. Participants were assigned to either the intervention group (*n* = 26) or the control group (*n* = 26) at the Obstetrics and Gynecology Clinic of Police General Hospital. Cord blood PRP was applied to the subcutaneous layer and the surgical wound immediately following the cesarean section. The efficacy of wound healing was evaluated using the REEDA scale score on days 1 and 3 postoperatively, and the Vancouver Scar Scale (VSS) was assessed in the 8th week postoperation. The efficacy in reducing pain was measured using a Visual Analog Scale on days 1 and 3 postoperatively. The mean REEDA scale on day 1 (mean ± SD: 1.5 ± 0.2561.5 ± 0.256 in the CB-PRP group and 2.5 ± 0.267 in the control group; *p*=0.009) and the mean VSS score at the 8th week (mean ± SD: 2.577 ± 2.003 in the CB-PRP group and 6.962 ± 2.441 in the control group; *p* < 0.001) were significantly lower in the CB-PRP group than those in the control group. However, there were no differences in Visual Analog Scale values between the two groups. The findings indicate that CB-PRP potentially promotes wound healing following cesarean sections but does not reduce pain. Further research is needed to confirm the beneficial effects of CB-PRP.

## 1. Introduction

Cesarean section, a surgical procedure for delivering a baby through an incision made in the mother's abdomen, is generally employed when serious problems prevent vaginal delivery. While cesarean delivery is relatively safe, it still carries risks such as infection, blood loss, internal organ injury, and complications from anesthesia [1]. Surgical scars and wound pain are also common issues associated with this procedure. Most women experience pain for the first few days after a cesarean, and for some, the pain can persist for several weeks.

**Figure 1:**
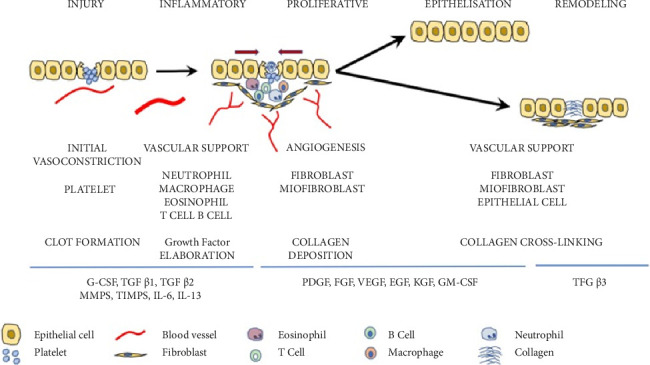
Wound-healing process.

Wound healing involves a complex process aimed at restoring the barrier. During this process, the migration, infiltration, proliferation, and differentiation of various cell types, including keratinocytes, fibroblasts, endothelial cells, macrophages, and platelets, occur, resulting in inflammation, proliferation, and tissue remodeling [2]. A complex signaling network involving numerous growth factors and cytokines plays an important role in regulating this complex process (Figure 1) [2].

**Figure 2:**
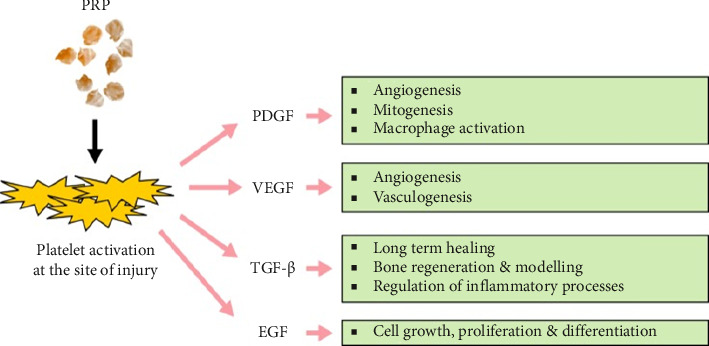
Mechanism of action of PRP in wound healing and tissue regeneration by various growth factors produced from platelets [3].

Platelet-rich plasma (PRP) is centrifuged fresh human blood with high concentrations of platelets, containing many growth factors, cytokines, and anti-inflammatory factors, including epidermal growth factor (EGF), vascular endothelial growth factor (VEGF), fibroblast growth factor (FGF), insulin-like growth factor-1 (IGF-1), and tumor necrosis factor-alpha (TNF-α) interleukins and interferons (Figure 2) [4]. These growth factors and cytokines are believed to stimulate natural healing mechanisms, repair damaged tissue, and restore normal function; therefore, they accelerate the wound-healing process and may relieve pain at the site of PRP injection [5].

Since the 1970s, PRP has garnered attention in various medical fields, such as in the treatment of diabetic foot ulcers [6], acute muscle injuries [7], dentistry [8], and dermatology [9]. The clinical application of PRP has expanded to gynecological disorders such as refractory endometrium, Asherman's syndrome, poor ovarian response, female sexuality, and urinary incontinence [10]. It has been introduced to reduce the risk of wound dehiscence, wound infection, and scar formation [11, 12] and to improve wound healing in patients undergoing cesarean sections [13].

Although PRP has demonstrated a significant positive effect on cesarean section wounds [13], there is a lack of literature reporting on the use of cord blood platelet-rich plasma (CB-PRP) for treatment on surgical wounds. CB-PRP possesses better properties compared to PRP in promoting wound healing and relieving pain due to higher levels of growth factors and anti-inflammatory molecules, which are crucial for the growth, proliferation, and differentiation of cells in fetal blood [14, 15]. Clinical evidence has shown encouraging results in wound healing following CB-PRP treatment for dermatological disorders [16] and diabetic foot [17]. Given its properties for wound healing described above, it is plausible that CB-PRP is not inferior to PRP in terms of wound healing and is easier to obtain following delivery. We hypothesize that the injection of CB-PRP into surgical sites could improve wound healing and reduce pain in women undergoing cesarean sections.

## 2. Methods

### 2.1. Objective

#### 2.1.1. Primary Objective

To assess the effect of CB-PRP on wound healing following cesarean section.

#### 2.1.2. Secondary Objective

To assess the effect of CB-PRP on pain reduction following cesarean section.

#### 2.1.3. Characteristics of the Participants

This study was approved by the Research Ethics Committee of Police General Hospital, Bangkok, Thailand. Ethical approval was granted on December 27, 2022. The study was conducted at the Department of Obstetrics and Gynecology, Police General Hospital, Bangkok, Thailand. Written informed consent was obtained from all participants prior to recruitment. This trial was registered in the Thai Clinical Trials Registry (TCTR) on March 22, 2023.

From April 1, 2023, to October 30, 2023, all pregnant women undergoing cesarean sections under general anesthesia with morphine who agreed to participate were recruited at the Department of Obstetrics and Gynecology, Police General Hospital. Exclusion criteria included women with the following characteristics: (1) Rh-negative blood group to avoid Rh incompatibility, (2) hemoglobin < 7 mg/dL (severe anemia), (3) history of abnormal blood clotting, (4) platelet count < 150,000 cell/mm^3^, and (5) loss to follow-up at 8 weeks postpartum. Data are provided in Supporting section.

### 2.2. Interventions

**Figure 3:**
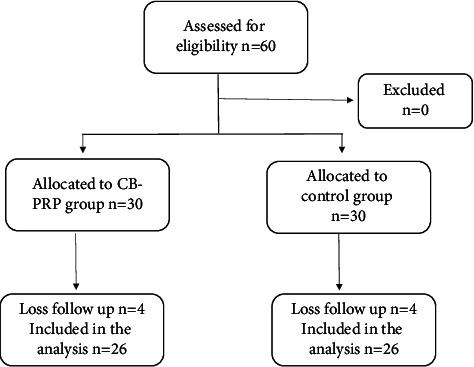
Flowchart of allocated participants.

This study is a randomized controlled trial. Participants were randomized into two groups: (1) intervention group and (2) control group Figure 3. The randomization schedule was generated using computer-generated random numbers prior to the cesarean section in accordance with the CONSORT 2010 checklist [18]. For the intervention group, CB-PRP was applied to the subcutaneous layer (4 mL) and the surgical wound (1 mL) immediately following the cesarean section, while no CB-PRP was applied to the control group. Subsequently, the skin was closed with intracutaneous Vicryl 2-0, and a wound dressing with a compression bandage was applied following skin closure. The participants were evaluated by physicians who were blinded to group allocation in the postpartum ward. Medical records contained no information indicating the group to which patients were allocated. Wound-healing efficacy was evaluated using the REEDA Scale on days 1 and 3 postoperatively, and the Vancouver Scar Scale (VSS) was assessed in the 8th week after the operation. The efficacy in reducing pain was measured using a Visual Analog Scale (VAS) on days 1 and 3 postoperatively. Images of surgical wounds were collected in the database for re-evaluation.

The REEDA scale assesses five elements of wound healing: redness, edema, ecchymosis, discharge, and approximation of the wound edges. Each element is rated on a scale of 0–3, with total scores ranging from 0 to 15. A lower REEDA score indicates better wound healing [19].

The VSS evaluates four variables: vascularity, height/thickness, pliability, and pigmentation, with a total score ranging from 0 to 14 [20]. The VAS evaluates pain by asking participants to indicate a degree of pain ranging from 0 to 100, with a higher VAS score indicating greater pain intensity [21].

### 2.3. PRP Preparation

**Figure 4:**
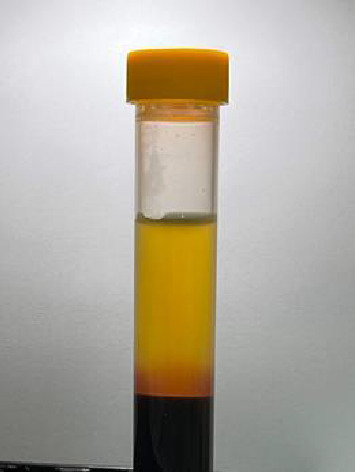
Separation of red blood cells (lower layer) from plasma containing platelets (upper layer) following low-speed centrifugation (250 g).

After the baby is born, blood is drawn from the umbilical cord (30 mL). The umbilical cord blood is divided into two plasma and growth factors (PP&GFs) tubes containing 1.5 mL of acid citrate dextrose (ACD) to prevent coagulation (12.5 mL each tube). Low-speed centrifugation (250 G) is performed for 12 min to separate red blood cells from plasma containing platelets (Figure 4) [22]. The portion of plasma containing platelets is aspirated and then mixed with calcium chloride in the proportion of 10:1 [22]. Subsequently, high-speed centrifugation (1000 G) is performed for 3 min to separate the plasma with a high platelet concentration (lower layer) from plasma with a low platelet concentration (upper layer). Plasma with a low platelet concentration is aspirated and discarded [22].

**Figure 5:**
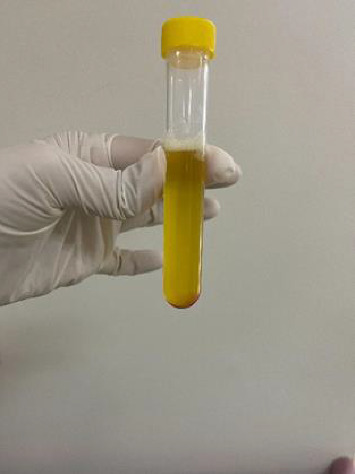
High concentration of platelets used for the subcutaneous layer and surgical wound injection.

With this centrifugation protocol, an average concentration equal to 2.5 times that of precentrifugation is achieved, which has been found to stimulate cell proliferation [23]. The concentration of CB-PRP was confirmed through testing (Figure 4). CB-PRP (Figure 5, 5 mL) was mixed and applied to the subcutaneous layer (4 mL) and the surgical wound (1 mL) in the intervention group. CB-PRP was prepared according to the protocol by two well-trained investigators to minimize variability.

### 2.4. Statistical Analysis

All statistical analyses were performed using a standard software package (STATA Stata Corp, Version 12.0). The minimum sample size was calculated using a *t*-test for two independent means based on a preliminary study by Tehranian et al. [13] investigating the effect of PRP on wound healing in cesarean section wounds (*m*1 = 1.85; m21.34; sd1 = 0.61; sd20.59; *α* = 0.05; and power = 0.8). Missing data were excluded from the analysis. Values were expressed as mean ± standard deviation (SD) and percentage as appropriate. Differences between groups were evaluated using an independent *t*-test for REEDA Score, VSS, and VAS. Associations between two categorical variables were determined using the Chi-square test.

## 3. Results

**Figure 6:**
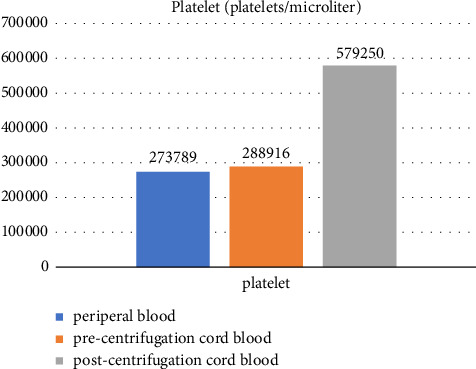
Platelet counts pre- and postcentrifugation.

**Figure 7:**
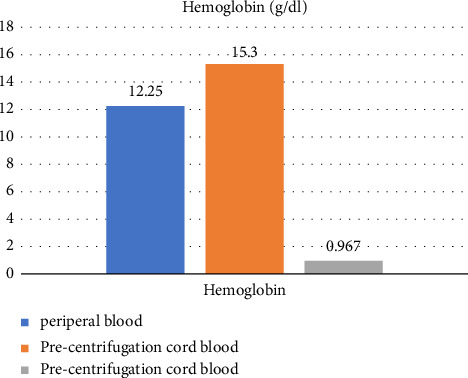
Hemoglobin levels pre- and postcentrifugation.

A total of 52 participants were eligible for recruitment and were allocated into the intervention group (*n* = 26) and the control group (*n* = 26) (Figure 3). There were no differences in baseline characteristics between the two groups (Table 1). General health conditions that could potentially affect the wound healing process, such as previous history of cesarean section, gestational diabetes mellitus (GDM), and anemia, were not statistically different between the two groups (Table 1). The duration of surgery and the amount of blood loss during surgery were not significantly different between the two groups (Table 2). There were no difference in hemoglobin levels (g/dL) and platelet counts (platelets per microliter) between the two groups prior to the operation (Table 2). After cord blood was centrifuged, the platelet counts (platelets per microliter) increased 2.03 times (from 288,916 to 579,250) Figure 6, while hemoglobin levels markedly decreased from 15.3 to 0.97 (g/dL) (Figure 7). The study group had a statistically lower REEDA score on postoperative day 1 than the control group, but the REEDA score was not significantly different on postoperative day 3 (Table 3). There were no difference between the two groups in VAS on both postoperative days 1 and 3 (Table 3). The VSS was significantly lower in the study group compared with the control group at postoperative week 8 (Table 3). Raw data lists are provided in Supporting 1.

## 4. Discussion

This study demonstrates that the application of CB-PRP in cesarean section wounds potentially improves wound healing and scar formation, as evidenced by a significant reduction in REEDA score and VVS at days 1 and 8 weeks. Thus, it could be implied that CB-PRP enhances healing at the surgical site shortly after surgery and positively affects skin restoration at least 2 months later. The effect is attributed to various growth factors and cytokines that play a crucial role in every phase of the wound-healing process, along with increased fibroblast activity [24–26]. Confounding factors that could affect the healing process, including the number of patients with previous cesarean sections, GDM, and anemia, were comparable following randomization between the two groups, thereby strengthening the reliability of the statistical comparisons. Furthermore, the skin incision was reapproximated using subcuticular sutures for both primary and secondary cesarean sections to avoid technical differences that could affect the wound-healing process.

It is well established that PRP promotes wound healing [27]. In recent years, the application of PRP to surgical wounds has become more frequent, aiding in wound healing [28–30] and decreasing pain [31–33]. It has been effectively applied in various types of wounds, including burns [34], acute/chronic wounds [35, 36], and surgical wounds [37, 38]. A small study by Nazari et al. found that PRP is effective in aiding wound healing in women undergoing Pfannenstiel incisions in gynecological surgeries, which is comparable with our study [37]. Alser et al. demonstrated improvements not only in healing parameters but also in early scar quality following cesarean section [39]. CB-PRP exhibits similar properties to PRP in adults [40] but contains higher levels of growth factors and cytokines [41]. Although several in vitro studies have shown the promising effects of CB-PRP in accelerating the healing process [40, 42, 43], clinical applications of CB-PRP remain limited. To the best of our knowledge, this study is the first to introduce the advantageous effects of CB-PRP on cesarean section wounds. Current data regarding CB-PRP and wound healing are conflicting. A study by Foffa et al. assessed the efficacy of CB-PRP in 10 patients with chronic ulcers and found a significant reduction in wound size after treatment [44]. However, another randomized clinical trial demonstrated no significant differences in wound size in patients with diabetic foot ulcers (*n* = 30) [45]. Notably, both studies recruited small sample sizes, making it difficult to draw definitive conclusions.

While pain reduction is theoretically possible due to the wound-healing properties of CB-PRP, the mechanism of pain relief is not well understood [46]. The result of this study indicates that CB-PRP enhances the wound-healing process and reduces scar formation; however, it does not alleviate pain. Current data regarding pain relief remain controversial. Some studies have demonstrated the positive effects of PRP on reducing pain at surgical sites [13, 31], whereas some studies did not [37]. Fanning et al. and Tehranian et al. showed that VSS was significantly lower in patients with PRP application undergoing gynecologic surgery [31] and cesarean surgery [13], respectively. On the contrary, a recent study by Nazari et al. found that women who gave birth by cesarean and received PRP had a nonsignificant difference in VSS in comparison with the control group [37]. Thus, further research is required to elucidate the actual mechanism of PRP in pain reduction and its clinical applications.

This study is the first randomized controlled trial utilizing CB-PRP to improve wound healing at the surgical site of cesarean sections. The randomized research methods limit selection bias of subjects between the interventions. Autologous cord-blood PRP is easy to collect and use during the operation; moreover, it is relatively inexpensive and safe. The results of this study could serve as preliminary data for designing future randomized controlled trials to ensure the beneficial effects of CB-PRP on cesarean section incision sites. No studies have reported the use of CB-PRP in patients undergoing cesarean sections. The most relevant studies have shown the positive effects of PRP on cesarean section wounds [13]. Therefore, it is intriguing to compare the efficacy of CB-PRP and PRP and other scar prevention methods (e.g., silicone gel and silicone scar sheet).

The limitations of this study include the small sample size of 52 participants, making it challenging to conclude definitively that CB-PRP is effective in improving wound healing and reducing scar formation. Furthermore, the study was not double blind and had a short-term follow-up period (8 weeks for VVS), which is relatively insufficient for scar assessment, as the remodeling stage begins and continues for several weeks to year [39]. We suggest that future double-blinded studies extend the evaluation of scars up to 1 year with a larger sample size. More objective tools, such as a cutometer (for assessing skin elasticity), a chromameter (for assessing skin color), and ultrasound (for assessing wound thickness), may be employed for better objective assessment. Collectively, the beneficial effects of PRP injection are promising but require further supporting evidence.

## 5. Conclusions

CB-PRP is an innovative therapeutic modality with a promising effect on improving wound healing and scar formation. It is affordable, simple, easy to perform, and has no serious adverse effects; however, due to the limited number of studies, more research is needed to confirm the beneficial effects of CB-PRP in the future.

## Figures and Tables

**Table 1:** Demographic data.

Baseline characteristics	Intervention group (*n* = 26)	Control group (*n* = 26)	*p* value
Age (mean ± SD)	29.077 ± 1.126	28.308 ± 1.138	0.653
BMI	23.954 ± 1.13	27.075 ± 1.433	0.092
Previous cesarean section	8	3	0.09
GDM	6	5	0.734
Anemia	8	11	0.388

**Table 2:** Baseline complete blood count (CBC), operation time, and blood loss.

	All participants	Intervention group	Control group	*p* value
Hemoglobin (mean ± SD)	12.254 ± 0.185	12.262 ± 0.253	12.246 ± 0.275	0.967
Platelet count (mean ± SD)	273,789 ± 8.341	265,615 ± 12.533	281,962 ± 11.019	0.332
Operating time (mean ± SD)	60.78 ± 1.696	62.038 ± 2.866	59.5 ± 2.161	0.483
Blood loss (mean ± SD)	439.423 ± 30.187	467.308 ± 44.974	411.539 ± 40.412	0.361

**Table 3:** The difference of Reeda, Vancouver Scar Scale, and Visual Analog Scale between the intervention and control groups.

	Study group		Control group		*p* value
	*n*	Mean ± SD	*n*	Mean ± SD	
REEDA score (day 1)	26	1.5 ± 0.256	26	2.5 ± 0.267	0.009
REEDA score (day 3)	26	0.923 ± 2.6	26	1.115 ± 2.171	0.572
VAS (day 1)	26	3.731 ± 0.439	26	4.192 ± 0.464	0.473
VAS (day 3)	26	2.884 ± 0.333	26	2.885 ± 0.33	0.624
VSS (week 8)	26	2.577 ± 2.003	26	6.962 ± 2.441	< 0.001

Abbreviations: VAS, Visual Analog Scale; VSS, Vancouver Scar Scale.

## Data Availability

Lists of raw data are provided in Supporting 1.

## Supporting Information

Raw data lists are provided in Supporting 1.
