# Supplementary material for: Cord Blood Platelet-Rich Plasma in Cesarean Section Wound Management
Source: Obstet Gynecol Int. 2024 Dec 12;2024:4155779. doi: 10.1155/ogi/4155779 (PMC11658848; doi:10.1155/ogi/4155779)
Supplement: Supplementary file 1 [file 4155779.f1.docx]

intervention	studyid	hn	age	agecat	complete	weigth	BMI	bmicat	prepregnancywt	height	prepregbmi	pre-op BMI	ud	G	P	GA	birthweight	previouscscat	dm	twin	steroid	hb	Hb	anemia	plt
1	2	640042115	28	2	1	66.6	27.72112383	4	54	1.55	24	3	none	1	0	2	2740	0	0	0	0	0	11.4	0	355
1	3	640052202	36	3	1	86	34.44960743	5	76	1.58	30.44	5	none	3	2	2	2970	0	1	0	0	0	13.4	0	261
1	4	531439869	34	2	1	60.9	24.39512899	3	55	1.58	22.03	2	none	3	1	2	3710	0	0	0	0	0	11.9	0	203
1	5	649022600	24	2	1	62.8	23.06703398	3	47	1.65	17.26	1	none	1	0	2	3230	0	0	0	0	0	12.8	0	236
0	6	650019150	20	2	1	64.4	28.24437525	4	56	1.51	24.56	3	none	1	0	2	2510	0	0	0	0	0	10.2	1	287
0	7	620025227	21	2	1	88	36.16042078	5	78	1.56	32.47	5	none	1	0	1	2515	0	1	0	0	0	11.7	0	255
1	8	640052781	21	2	1	57.1	23.16523997	3	51	1.57	20.7	2	Thalassemia	1	0	2	3110	0	0	0	0	0	9.1	1	359
1	9	640053017	38	3	1	92	37.80407627	5	79	1.56	32.9	5	none	3	2	2	3785	0	1	0	0	0	13.6	0	196
1	10	640050998	20	2	1	86.1	35.83766909	5	65	1.55	27.1	4	none	3	0	1	2350	0	0	0	0	0	12.4	0	386
0	11	640054721	23	2	1	50	19.53125	2	44.5	1.6	17.38	1	none	3	2	1	2599	1	0	0	0	0	13.1	0	236
0	12	531494672	29	2	1	92.7	34.04958678	5	73	1.65	28.99	4	none	2	1	1	3300	0	0	0	0	0	12.6	0	342
0	13	531105451	35	3	1	67.7	25.7963725	4	56	1.62	21.34	2	none	4	2	2	3190	1	0	0	0	0	11	0	208
0	14	640050991	26	2	1	92.7	34.46609161	5	89	1.64	31.53	5	none	2	1	2	3150	0	0	0	0	0	9.6	1	293
0	15	610033234	25	2	1	120	46.875	5	120	1.6	46.88	5	none	3	2	2	3640	1	0	0	0	0	10.9	1	306
1	16	640051293	37	3	1	101	43.14579862	5	87	1.53	37.17	5	none	1	0	1	2670	0	1	0	0	0	12.8	1	233
0	18	650017185	32	2	1	81.7	32.72712706	5	72	1.58	29.97	4	none	1	0	2	2960	0	0	0	0	0	14.6	1	221
1	19	650022981	31	2	1	54	21.63114885	2	54	1.58	21.88	2	none	4	1	2	3080	1	1	0	0	0	12.5	1	247
0	21	600025059	32	2	1	94.5	33.08707678	5	93	1.69	32.56	5	none	1	0	2	3240	0	1	0	0	0	11.4	1	259
1	22	640050276	27	2	1	73.6	31.03390116	5	52	1.54	21.92	2	none	1	0	2	2600	0	0	0	0	0	14.3	1	229
1	23	531592696	27	2	1	65.8	29.24444444	4	50	1.5	22.22	2	none	1	0	2	3090	0	0	0	0	0	13.7	1	335
0	24	640049154	25	2	1	107	47.55555556	5	89	1.5	37.53	5	none	1	0	2	2380	0	0	0	0	0	13.7	1	331
1	25	590016759	25	2	1	62.9	26.87000726	4	53	1.53	22.64	2	none	2	1	2	3110	1	0	0	0	0	9.8	0	405
0	26	580035612	22	2	1	51	22.66666667	2	39	1.5	17.33	1	none	1	0	2	2740	0	0	0	0	0	12	1	270
0	27	650022670	20	2	1	60.6	26.93333333	4	50	1.5	20.53	2	none	1	0	2	2485	0	0	0	0	0	14.7	1	270
0	29	60002487	30	2	1	69.2	25.41781451	4	55	1.65	20.2	2	none	2	1	2	2580	0	0	0	0	0	14	1	253
1	31	650021652	31	2	1	102.1	39.8828125	5	85.1	1.6	33.2	5	none	1	0	2	3200	0	0	0	0	0	11.3	1	275
0	32	620028510	35	3	1	75.7	33.64444444	5	67	1.5	29.78	4	none	2	0	2	2530	0	1	0	0	0	14.6	1	455
1	33	650017054	30	2	1	62.8	26.13943809	4	46	1.55	19.56	2	none	1	0	2	3210	1	0	0	0	0	12	1	226
1	34	650023853	27	2	1	75.8	27.17917458	4	56	1.67	20.08	2	none	4	3	2	3210	1	0	0	0	0	12	1	250
1	35	640055290	33	2	0	64.8	25.3125	4	52	1.6	20.31	2	anemia	2	1	2	2890	1	0	0	0	0	10.9	0	166
0	36	531556974	20	2	1	108.8	41.45709496	5	100	1.62	38.1	5	none	1	0	2	3270	0	0	0	0	0	12.5	0	355
1	37	610005309	28	2	1	63.3	24.7265625	3	44	1.6	17.19	1	none	2	1	2	3620	0	0	0	0	0	12.4	0	273
1	38	640053670	32	2	1	58.4	24.9476697	3	45	1.53	19.22	2	none	2	1	2	3345	1	0	0	0	0	14.1	0	266
1	39	630015303	21	2	1	83.1	35.03963569	5	78	1.54	33.32	5	none	4	0	2	3460	0	0	0	0	0	11.6	0	179
0	41	610000473	37	3	1	84	30.11940191	5	74	1.67	26.6	4	none	2	1	2	3430	0	0	0	0	0	13.2	0	218
1	45	531491848	36	3	1	47	20.88888889	2	45	1.5	20	2	none	1	0	1	2105	0	0	0	0	0	11.1	0	314
0	47	640053742	27	2	1	51	19.9	2	44	1.6	17.9	1	none	1	0	2	2866	0	0	0	0	0	11.3	0	225
1	48	650019647	17	1	1	54	23.37257618	3	43	1.52	18.61	2	none	1	0	2	2260/2210	0	0	1	0	0	14.1	0	296
0	49	531234781	33	2	0	64.8	26.97190427	4	48	1.55	19.97	2	none	3	2	2	3030	0	0	0	0	0	12.7	0	245
0	50	650023137	24	2	1	63.2	28.8531775	4	53	1.48	24.19	3	none	1	0	2	3090	0	0	0	0	0	12.6	0	272
0	51	640057858	24	2	1	81.3	36.13333333	5	78	1.5	34.67	5	none	1	0	2	3230	0	1	0	0	0	12.3	0	349
0	52	531456020	37	2	1	70.8	28.72327478	4	50	1.57	20	2	none	5	2	2	3890	0	1	0	0	0	11.9	0	312
1	54	531485318	37	3	1	74	28.90625	4	57	1.6	22.6	2	none	2	1	2	3150	0	1	0	0	0	12.7	0	221
0	56	540000226	29	2	1	91	33.83402736	5	74	1.64	27.51	4	none	2	1	2	4280	0	0	0	0	0	11.7	0	266
0	57	650014052	33	2	1	73	30.38501561	5	50	1.55	21	2	none	2	1	2	2540	0	0	0	0	0	9.8	1	302
0	61	620041689	37	3	1	79.5	29.20110193	4	75	1.65	27.55	4	Myoma uteri	1	0	2	3640	0	0	0	0	0	11.6	0	223
0	62	650023151	25	2	1	83.2	33.75390482	5	73	1.57	29.62	4	none	2	1	2	2910	0	0	0	0	0	13.2	0	240
0	63	650010045	35	3	1	72.1	30.01040583	5	62	1.55	25.8	4	none	2	0	2	2465	0	0	0	0	0	11.5	0	338
1	64	650015007	25	2	1	54.3	22.60145682	3	42	1.55	17.48	1	none	1	0	2	3350	0	0	0	0	0	13.6	0	178
1	65	650013824	43	3	1	76	29.6875	4	60	1.6	23.44	3	none	3	2	2	3530	1	0	0	0	0	11.4	0	254
1	66	610004072	25	2	1	84.9	34.88658777	5	64	1.56	26.29	4	none	2	1	2	3270	1	0	0	0	0	11.4	0	256
1	67	650029889	23	2	1	97.5	37.15134888	5	82	1.62	31.25	5	none	1	0	2	3580	0	1	0	0	0	12.5	0	307
				1= teenage												preterm= 1		previous c/s= 1	Dm= 1	twin=1					
				2= normal			< 18= 1									term= 2		none= 0	none= 0	none=0					
				3= elderly			18.5-22.9= 2									postterm= 3									
							23-24.9= 3																		
							25-29.9= 4																		
							> 30= 5																		

intervention	indicatio	prom	optime	ebl	surgeon	REEDA c	VSS Day 1	VAS Day 1	VAS d1 cat	REEDA Day 3	VSS Day 3	VAS Day 3	VAS d3 cat	REEDA we	VSS Week	VAS Week	VAS wk8 cat	ivanalgesia	chb	chct	cplt	phb	phct	pplt	pwbc	increase plt
1	2	0	51	300	1	1	0	2	1	0	0	1	1	0	5	0	1	0	15.8	47.7	238000	0.7	0.9	442000	3000	1.8571429
1	6	0	85	500	0	1	0	2	1	0	0	0	1	0	4	0	1	1	14.3	40.8	324000	0	0.5	309000	100	0.9537037
1	1	0	52	300	1	1	0	2	1	1	0	5	2	0	5	0	1	2	16.3	47.8	254000	0.4	0.1	600000	100	2.3622047
1	6	1	65	800	0	3	0	5	2	2	0	4	2	0	3	0	1	0	14.6	42	319000	0.6	0.7	592000		1.8557994
0	2	0	48	300	1	3	0	2	1	2	0	4	2	0	11	0	1	1								
0	1	1	62	700	0	5	0	2	1	4	0	3	1	0	4	1	1	0								
1	2	0	60	300	0	4	0	2	1	0	0	6	3	0	2	0	1	0								
1	1	0	90	700	0	2	0	7	3	0	0	2	1	0	0	0	1	0	16.1	49.5	257000	0.4	0.7	405000	1600	1.5758755
1	6	0	60	400	0	1	0	4	2	0	0	3	1	0	0	0	1	0								
0	3	0	72	300	0	1	1	1	1	1	0	2	1	0	11	0	1	1								
0	5	1	55	400	0	4	0	1	1	3	0	3	1	0	11	1	1	0								
0	3	0	70	500	0	5	0	8	3	2	0	5	2	0	10	0	1	0								
0	1	0	48	600	0	1	0	3	1	1	0	3	1	0	6	0	1	1								
0	3	0	90	50	1	4	0	7	3	2	0	4	2	1	4	0	1	0								
1	6	0	60	400	0	3	0	1	1	0	0	3	1	0	2	0	1	0	14.9	42.5	290000	1	1.8	367000	1700	1.2655172
0	6	0	65	600	0	3	0	5	2	1	0	1	1	0	7	0	1	0								
1	3	0	60	400	0	0	0	8	3	0	0	0	1	0	5	0	1	4								
0	6	0	45	400	0	4	0	1	1	1	0	1	1	0	6	0	1	0								
1	1	0	40	300	0	1	0	1	1	1	0	2	1	0	3	0	1	0								
1	1	0	60	500	0	0	0	4	2	0	0	1	1	0	1	0	1	0								
0	6	0	70	400	0	3	0	4	2	3	0	3	1	0	7	0	1	0								
1	3	0	100	1000	1	1	0	5	2	1	0	0	1	0	5	0	1	3								
0	1	0	61	600	0	1	0	6	3	0	0	2	1	0	8	0	1	1								
0	1	1	43	300	0	3	0	4	2	2	0	2	1	0	5	0	1	0								
0	6	0	58	300	0	1	0	4	2	1	0	2	1	0	9	0	1	0								
1	1	0	54	400	0	1	0	2	1	1	0	2	1	0	1	0	1	1	14.4	40	324000	0.8	0.9	642000	1600	1.9814815
0	5	1	67	250	0	1	0	6	3	0	0	2	1	1	4	0	1	1								
1	2	0	63	250	0	2	0	2	1	1	0	1	1	0	1	0	1	0								
1	3	0	63	400	0	2	0	4	2	0	0	2	1	0	1	0	1	0	10.3	37.2	8900	2.9	6.8	53000	4500	5.9550562
1	3	0	60	300	0	3	0	7	3	2	0	2	1	0	3	0	1	0								
0	1	0	60	300	1	2	0	1	1	0	0	1	1	0	9	0	1	2								
1	2	0	50	300	0	1	0	2	1	1	0	2	1	0	1	0	1	0								
1	3	0	90	1200	1	5	0	2	1	5	0	4	2	0	1	0	1	2								
1	6	0	60	400	0	1	0	2	1	0	0	1	1	0	0	0	1	0								
0	6	0	70	700	0	1	0	2	1	0	0	2	1	0	7	0	1	1								
1	2	0	60	300	0	0	0	4	2	0	0	4	2	0	1	0	1	2								
0	2	0	48	200	0	2	0	5	2	1	0	3	1	0	8	0	1	0								
1	4	0	60	600	0	3	0	7	3	1	0	3	1	0	6	0	1	2	16.9	47.6	381000	1	0.6	1120000	5200	2.9396325
0	6	0	50	100	0	3	0	9	3	1	0	4	2	0	6	0	1	2								
0	1	0	60	800	0	4	0	2	1	0	0	1	1	0	8	0	1	0								
0	1	0	65	300	0	2	0	4	2	0	0	5	2	0	7	0	1	1								
0	6	0	60	400	0	3	0	3	1	0	0	2	1	0	3	0	1	1								
1	6	0	70	500	0	1	0	6	3	1	0	3	1	0	1	0	1	3	15.5	44	258000	0.9	0.8	602000	3100	2.3333333
0	1	0	50	300	0	3	0	5	2	0	0	2	1	4	6	0	1	2								
0	5	1	53	200	0	3	0	4	2	1	0	2	1	0	8	0	1	1								
0	2	0	72	800	0	2	0	7	3	2	0	8	3	1	4	0	1	0								
0	2	0	60	600	0	0	0	5	2	1	0	2	1	0	9	3	1	0								
0	6	0	45	300	0	1	0	8	3	0	0	6	3	0	3	3	1	0								
1	1	0	53	300	0	1	0	8	3	4	0	4	2	0	3	0	1	0								
1	3	0	38	400	1	0	0	2	1	0	0	5	2	1	7	2	1	0	14.3	39.3	263000	1.4	2.4	782000	3500	2.973384
1	3	0	53	500	0	0	0	2	1	3	0	4	2	0	4	0	1	0	17.9	50.2	230000	3	6.6	720000	3100	3.1304348
1	1	0	56	400	0	1	0	4	2	0	0	5	2	0	2	0	1	1	13.3	37.1	329000	1.4	2.6	370000	3500	1.1246201
													mild= 1													
													modurate= 2													
													severe= 3													
